# Intradermal administration of magnesium sulphate and magnesium chloride produces hypesthesia to mechanical but hyperalgesia to heat stimuli in humans

**DOI:** 10.1186/1742-2094-6-25

**Published:** 2009-08-28

**Authors:** Takahiro Ushida, Osamu Iwatsu, Kazuhiro Shimo, Tomoko Tetsunaga, Masahiko Ikeuchi, Tatsunori Ikemoto, Young-Chang P Arai, Katsutoshi Suetomi, Makoto Nishihara

**Affiliations:** 1 Multidisciplinary Pain Center, Aichi Medical University, 21 Karimata, Yazako, Nagakute, Aichi 480-1195, Japan; 2 Department of Orthopaedic Surgery, Kochi Medical School, Kohasu, Okoh-cho, Nankoku, Kochi 783-8505, Japan; 3 Nankoku Pain Research Group, Kochi Medical School, Kohasu, Okoh-cho, Nankoku, Kochi 783-8505, Japan

## Abstract

**Background:**

Although magnesium ions (Mg^2+^) are known to display many similar features to other 2+ charged cations, they seem to have quite an important and unique role in biological settings, such as NMDA blocking effect. However, the role of Mg^2+ ^in the neural transmission system has not been studied as sufficiently as calcium ions (Ca^2+^). To clarify the sensory effects of Mg^2+ ^in peripheral nervous systems, sensory changes after intradermal injection of Mg^2+ ^were studied in humans.

**Methods:**

Magnesium sulphate, magnesium chloride and saline were injected into the skin of the anterior region of forearms in healthy volunteers and injection-induced irritating pain ("irritating pain", for short), tactile sensation, tactile pressure thresholds, pinch-pain changes and intolerable heat pain thresholds of the lesion were monitored.

**Results:**

Flare formation was observed immediately after magnesium sulphate or magnesium chloride injection. We found that intradermal injections of magnesium sulphate and magnesium chloride transiently caused irritating pain, hypesthesia to noxious and innocuous mechanical stimulations, whereas secondary hyperalgesia due to mechanical stimuli was not observed. In contrast to mechanical stimuli, intolerable heat pain-evoking temperature was significantly decreased at the injection site. In addition to these results, spontaneous pain was immediately attenuated by local cooling.

**Conclusion:**

Membrane-stabilizing effect and peripheral NMDA-blocking effect possibly produced magnesium-induced mechanical hypesthesia, and extracellular cation-induced sensitization of TRPV1 channels was thought to be the primary mechanism of magnesium-induced heat hyperalgesia.

## Background

Although magnesium ions (Mg^2+^) are widely distributed throughout the whole organ, the role of Mg^2+ ^in the neural transmission system has not been studied as sufficiently as calcium ions (Ca^2+^). Much research has mentioned that Mg^2+ ^shows a similar physiological attitude to Ca^2+ ^and it has been reported that both ions have a membrane-stabilizing effect on nerves [1,2]. In addition, Mg^2+ ^is known to act as a competitor to Ca^2+^, in extracellular matrix [3]. However, the specific role of Mg^2+ ^in neurophysiological transmission, especially concerning peripheral somatosensory systems, has been insufficiently focused on and not understood enough.

Among the various studies, the noncompetitive antagonistic action of Mg^2+ ^on N-methyl-D-aspartate (NMDA) receptor, a glutamate receptor, was the focus of various reports [4]. Although the role of spinally-located NMDA receptors has been the focus of pain-related research before, NMDA receptors are also known to exist in peripheral nervous system [5]. Carlton et al. reported an increased population of peripheral glutamate receptors in injured peripheral nerve tissue [6]. In a previous study, Iwatsu et al. reported that intradermal administration of NMDA receptor antagonists, ketamin hydrochloride and magnesium sulphate, produces hypesthesia to mechanical stimuli in humans [7]. Therefore, Mg^2+ ^may alter neuronal activities both centrally and peripherally.

Concerning therapeutic effects, magnesium sulphate is known to improve types of pain in humans and animals [8]. On the other hand, Mikkelsen et al. reported that intravenous injection of Mg^2+ ^(magnesium sulphate) produces heat hyperalgesia in humans [9].

Since much previous research has reported contradictory results, it is necessary to organize human investigation to clarify real changes of sensory experiences after administrations of Mg^2+^. In our healthy volunteer study, subjects were asked to estimate the degree of pain and the effect of the drug was examined. In addition to noxious mechanical stimulation and noxious radiant heat stimulation, we performed an experiment evaluating innocuous mechanical stimulation, such as tactile sensation, in order to investigate the effect of intradermally applied magnesium sulphate (MS) and magnesium chloride (MC).

## Methods

Fifteen healthy volunteers (age ranged from 26 to 34 years, mean: 29 years) were enrolled in sensory testing study after intradermal injection of magnesium ions and another 15 healthy volunteers (age ranged from 22 to 43 years, mean: 28 years) were enrolled in the experiment for examining the effect of local cooling in Mg^2+ ^ion induced irritating pain study. All protocols were conducted in accordance with the recommendations outlined in the Declarations of Helsinki and were approved by the local Medical Ethical Committee. All subjects agreed to the study protocols and signed an informed consent form prior to the examination.

### Sensory testing study after intradermal injection of magnesium ion

Using the double-blind method, each subject (n = 15) received one intradermal injection of 0.5 M MS (0.1 ml, 524 mOSM) into one anterior ulnar site on the forearm and one injection of physiological saline (0.1 ml, 0.9% NaCl, 290 mOSM) at the same site into the other forearm as a control. At least one week after injection of 0.5 M MS, the same subjects received one injection of 0.05 M MS (0.1 ml, 337 mOSM) and saline in the same way. With 0.05 M MC and physiological saline solutions, they were injected with MC (0.1 ml, 385 mOSM) and physiological saline (0.1 ml) under the same procedure at least one week later. Therefore, each subject received one injection of 0.5 M & 0.05 M MS, and 0.05 M MC, and three injections of saline. Following injection, the resulting effects were evaluated after 1, 10, 20, 30, 45 and 60 min. for the MS site, MC site and NaCl site. The following tests were undertaken in a quiet room and room temperature was maintained at 25°C. Skin surface around the injection (test) area was kept at 34°C by servo-controlled thermal controller (Dantec Dynamics, Skovlunde, Denmark).

### Injection-induced irritating pain evaluation

The intensity of irritating pain was evaluated by 100 mm visual analogue scale ('VAS') before and after injection of test solutions.

### Tactile sensation evaluation

Using a horse hair brush, tactile sensations at the wheal region formed by intradermal injection and the region of unaffected skin were compared. Rating the sense of normal skin on the same arm as 10, the tactile sensation at the wheal region was evaluated using a numeric rating scale ('NRS').

### Tactile pressure threshold test

Using the von Frey filaments, the tactile pressure threshold in intradermally injected area was measured. Prior to this experiment, pressure force of each von Frey filament was calibrated.

### Pinch-pain evaluation test

Using an arterial clamp, the pain intensity evoked by pinch at the wheal region formed by intradermal injection and unaffected normal proximal skin were compared. Rating the sense of pain of normal skin on the same arm as 10, the pain intensity at the wheal region was evaluated using NRS. In addition, same pinch (noxious mechanical) stimulations were applied to the skin, located 1 cm apart from wheal region to check existence of secondary hyperalgesia.

### Measurement of intractable heat pain evoking temperature 

Thermal stimulation was applied by Peltier probe controller (Intercross-200, Intercross Co., Tokyo, Japan). Tip of the probe (2.5 × 2.5 cm) was applied to injection site and temperature of the probe was serially increased from 30 to 50°C (+0.5°C/sec.). Experimental subjects were instructed to push a button when they experienced intolerant heat pain sensation (The threshold temperature that induces intolerable heat pain). After pushing the button, the temperature of the probe was programmed to automatically return quickly to 30°C.

### Changes in injection-induced irritating pain after cooling

**Figure 1:**
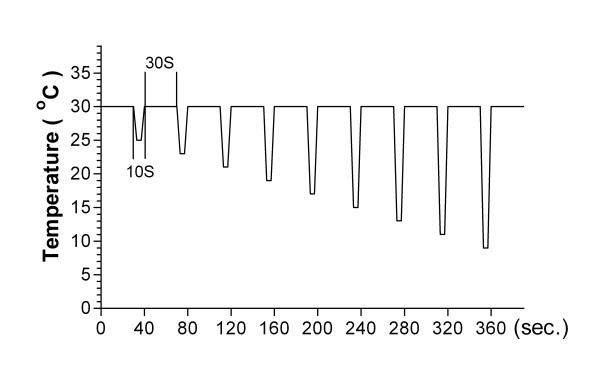
**Schematic diagram of the graded cooling stimuli**. To check changes in spontaneous pain appearing at the injection site, Peltier probe was directly attached to the injection site and cooled the skin in gradual increments.

After intradermal administration of MS (0.5 M), graded cooling stimuli (from 25 to 9°C: -0.5 – -2.1°C/sec) were applied to the injection site by Peltier probe controller (UDH-300, Unique Medical Co., Tokyo, Japan) (Fig. 1) and the intensity of irritating pain at each test temperature was recorded by NRS.

### Statistical Analysis

All data are expressed as mean ± S.E.M Significant changes over time were determined with the Friedman's analysis of variance by ranks followed by post hoc pairwise comparisons.

## Results

### Local observation and injection-induced irritating pain evaluation

**Figure 2:**
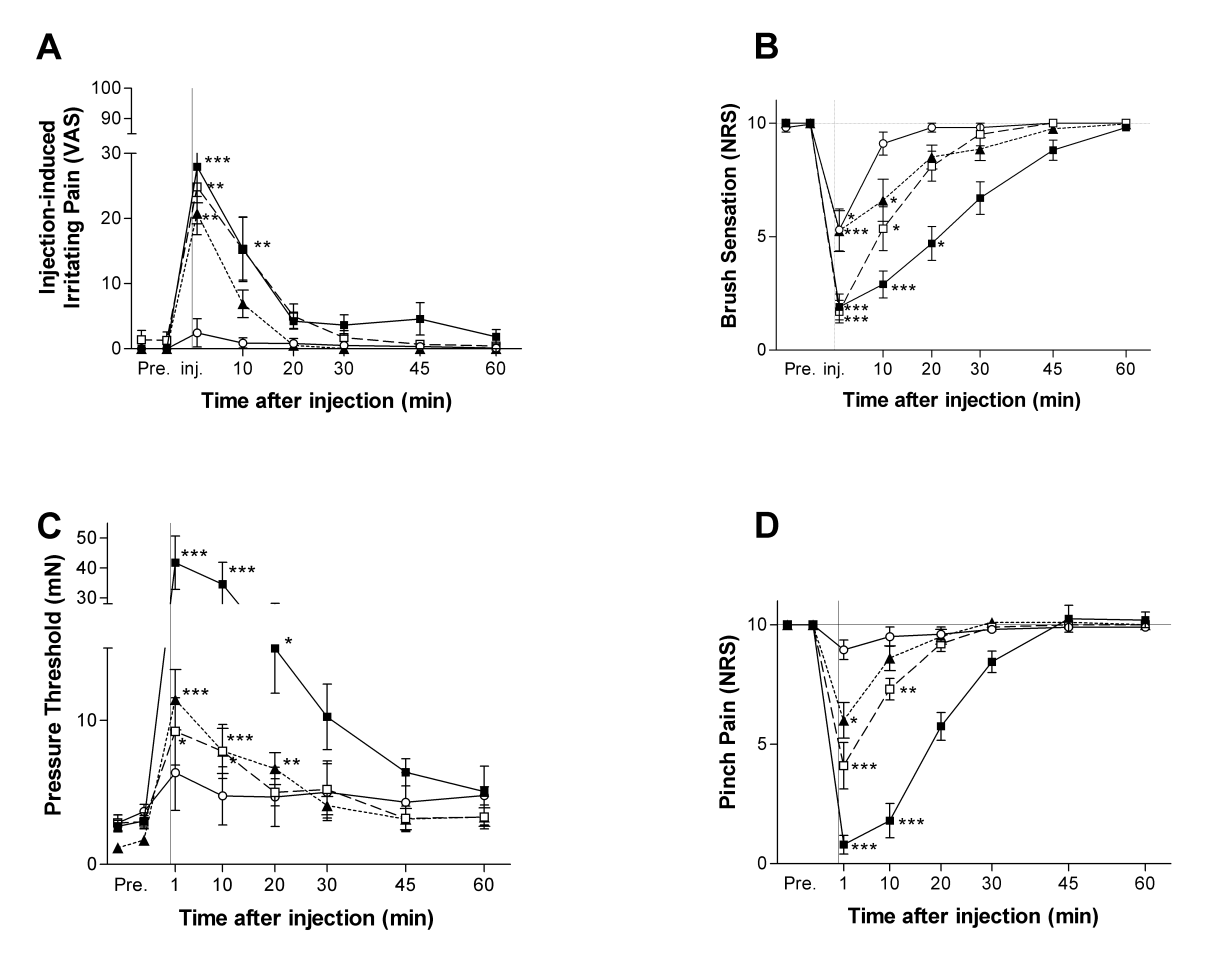
**Time course of changes in effect of magnesium ion on pain and sensations**. Fifteen volunteers were intradermally injected with 0.5 M MgSO_4 _(black square), 0.05 M MgSO_4 _(white square), 0.05 M MgCl_2 _(black triangle), or saline (white circle). Each volunteer was injected with three kinds of magnesium solution at intervals of at least one week. Local spontaneous pain (A) was reported by visual analogue scale (VAS), Tactile sensation (B) and pinch pain intensity (D) were reported by a numerical rating score (NRS). When MgSO_4 _and MgCl_2 _solutions were injected, transient irritating pain and local hypesthesia to mechanical stimuli appeared at the injection site. *p < 0.05 vs. control; **p < 0.01 vs. control, ***p < 0.001 vs. control. As values were similar among these three saline injections, we have put the representative data herein.

All test solutions containing Mg^2+ ^produced flare formation around the injection site and irritating pain. The intensity of irritating pain evaluated by VAS showed a significant increase at 1–10 min after the injection of 0.5 M and 0.05 M MS. Furthermore, MC produced irritating pain at 1 min after injection. (Fig. 2A)

### Tactile sensation evaluation

After injection of MS, tactile sensation caused by brush decreased up to 20 min and by 1–10 min after injection of 0.5 M and 0.05 M MS respectively, and by 1–10 min after injection of 0.05 M MC (Fig. 2B) compared with the control level.

### Tactile pressure threshold test

The tactile pressure threshold measured using the von Frey filaments significantly increased up to 20 min and by 1–10 min after injection of 0.5 M and 0.05 M MS respectively, and up to 20 min after injection of MC compared with saline. (Fig. 2C)

### Pinch-pain evaluation test

The pinch-pain evoked by an arterial clamp was reduced up to 10 min after injection of 0.5 M and 0.05 M MS. Similar but shorter changes were observed after injection of MC. (Fig. 2D)

In addition apparent secondary hyperalgesia was not detected in flare area.

### Measurement of intolerable heat pain evoking temperature

The threshold temperature that induces intolerable heat pain was decreased up to 20 min and by 1–10 min after intradermal injection of 0.5 M and 0.05 M MS respectively. Similarly MC decreased the pain evoking temperature up to 20 min after injection.

**Figure 3:**
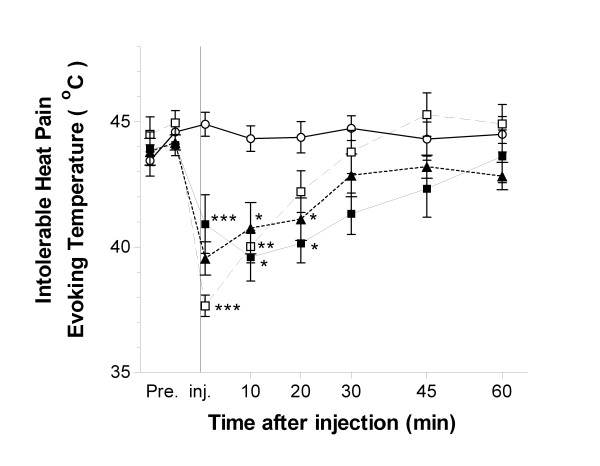
**Time course of changes in intolerable heat pain evoking temperature after intradermal injection of magnesium solution**. Fifteen volunteers were intradermally injected with 0.5 M MgSO_4 _(black square), 0.05 M MgSO_4 _(white square), 0.05 M MgCl_2 _(black triangle), or saline (white circle). Each volunteer was injected with three kinds of magnesium solution at intervals of at least one week. Intolerant heat pain temperature was decreased at least 10 min following local administration of Mg^2+^. *p < 0.05 vs. control; **p < 0.01 vs. control, ***p < 0.001 vs. control. As values were similar among these three saline injections, we have put the representative data herein.

In contrast, intradermal injection of saline did not alter the intolerable heat pain threshold. (Fig. 3)

### Effect of local cooling in Mg^2+ ^ion-induced irritating pain

**Figure 4:**
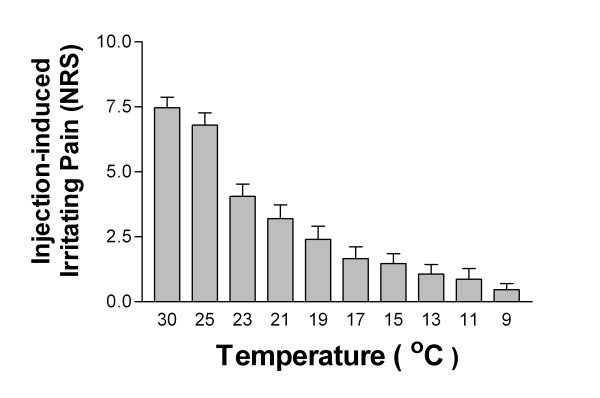
**Changes in experienced pain intensity by local cooling**. After intradermal administration of 0.5 M MgSO_4_, Peltier probe was directly attached to the injection site. Pain intensity was substantially attenuated according to the cooling temperature. (n = 15)

Cooling of the surface of the injected area apparently attenuated irritating pain. (Fig. 4) Furthermore, local flare did not change after local cooling.

## Discussion

In the present study, both MS and MC solutions but not saline, inhibited the sensations evoked by noxious and innocuous mechanical stimuli but produced irritating pain. Several possible mechanisms can explain the Mg^2+ ^ion-induced mechanical hypesthesia. As a general effect of the excitable membrane, changes in external divalent cations are considered to alter membrane surface potential and therefore, a high concentration of Mg^2+ ^may inhibit the excitability of the axon by raising the electrical threshold of the membrane directly [2,10]. In pain-related polymodal receptors, Sato et al. showed that reduction of extracellular Ca^2+ ^augmented the neuronal responses caused by hypertonic saline and high K solutions and also showed the augmented neuronal responses returned to control levels by an addition of Mg^2+^, which suggests Mg^2+ ^has a similar membrane-stabilizing effect on nerves to Ca^2+^[11].

In addition, Chaban indicated that NMDA receptors on the periphery are involved in the transmission of noxious mechanical stimulation [12]. Only little attention has being paid so far to the functional importance of the peripherally-distributed NMDA receptors [7]. In a previous study, Ushida et al. evaluated the importance of mechanical stimulation by studying the relationship between magnesium ions and peripheral NMDA receptors [13]. It was found that injection of MK-801, an NMDA receptor antagonist, into the peripheral skin of rats produces inhibition of the sensations induced by innocuous and noxious stimulation.

Paradoxically, present results have shown that local administration of Mg^2+ ^induces heat hyperalgesia, but mechanical hypesthesia at the injection site. Only a few researchers have paid attention to changes in Mg^2+ ^-induced thermal sensations. Oral administration [14] and intrathecal injection [15] of magnesium sulphate are reported to improve heat hyperalgesia in animal models of neuropathic pain. On the other hand, Mikkelsen et al. reported that intravenous infusion of magnesium had no analgesic effect on thermal sensation in hyperalgesic skin but produced a decreased heat detection threshold and increased pain caused by 1 min long 45°C heat stimulation [9]. Since we could not find any sensory changes outside of wheals, peripheral mechanisms were suggested to explain magnesium-induced heat hyperalgesia.

Recently, transient receptor potential cation channel, subfamily V, member 1 (TRPV1), a heat-sensitive ion channel, has been discovered [16] and the role of this channel may be implicated in Mg^2+^-induced heat hyperalgesia observed in our study. Indeed, Ahern et al. showed extracellular cations such as Na^+^, Ca^2+^, Mg^2+ ^modulate/open the gates of TRPV1 channel by in vitro whole cell and single channel patch-clamp recording studies [17]. In addition to this direct mechanism, several neuropeptides and inflammatory mediators may be implicated in the enhanced activation of TRPV1. The flare formation observed at the injection site suggesting existence of axonal reflex-induced neuropeptide (SP, CGRP, etc) release from sensory nerve endings and released SP may result in sensitizing TRPV1 via activation of NK1, a SP receptor [18]. Also these inflammatory processes may activate the production of bradykinin (BK), a novel algesic substance. Increased extracellular concentration of BK results in sensitization and activates TRPV1 currents via PLC, PKC, and lipoxygenase-derived products [19-21].

These previous studies suggest that TRPV1 may be implicated in Mg^2+^ -induced hyperalgesia. Since cooling is known to desensitize local TRPV1, attenuation of Mg^2+^ -induced irritating pain by local cooling observed in our present study may also be explained by this mechanism.

Recently, types of thermal-sensing receptors such as TRPM8 (Transient receptor potential cation channel, subfamily M, member 8: cool receptor), TRPA1 (transient receptor potential cation channel, member A1: cold receptor), etc. have been discovered after TRPV1 and have been widely investigated [22,23]. In clinical settings, various types of diseases and patients possess the symptoms of thermal (heat, cold) hyperalgesia. However, it has been problematic under such settings to specify the underlying pathological mechanisms in these patients. Presumably, expression or activation of these recently discovered thermal receptors may play a key role in thermal pain and further translational research is necessary for the understanding and treatment of these intractable pain syndromes.

## Conclusion

Intradermal administration of magnesium ions locally affected sensory systems and produced spontaneous pain, hypesthesia to both noxious and innocuous mechanical stimuli, and decreased the heat pain threshold. Activation of TRPV1 channel is the suggested mechanism for the development of heat hyperalgesia.

## Abbreviations

MS: magnesium sulphate; MC: magnesium chloride; NMDA: N-methyl-D-aspartate; TRPV1: transient receptor potential cation channel, subfamily V, member 1; SP: substance P; CGRP: calcitonin gene-related peptide; NK-1: neurokinin 1; BK: bradykinin; PLC: phospholipase C; PKC: protein kinase C; TRPM8: transient receptor potential cation channel, subfamily M, member 8; TRPA1: transient receptor potential cation channel, member A1

## Competing interests

The authors declare that they have no competing interests.

## Authors' contributions

TU conceived of the study, participated in its study, and conducted all experiments. OI, KS (Shimo) and TT conducted the acquisition of data. MI and TI performed the statistical analysis. YAP, MN and KS (Suetomi) helped to draft the manuscript. All authors read and approved the final manuscript.
